# High SARS-CoV-2 Seroprevalence among Healthcare Workers in Bamako, Mali

**DOI:** 10.3390/v14010102

**Published:** 2022-01-07

**Authors:** Anou M. Somboro, Yacouba Cissoko, Issiaka Camara, Ousmane Kodio, Mohamed Tolofoudie, Etienne Dembele, Antieme C. G. Togo, Djibril M. Ba, Yeya dit Sadio Sarro, Bocar Baya, Seydou Samake, Ibrahim B. Diallo, Alisha Kumar, Mohamed Traore, Bourahima Kone, Amadou Kone, Bassirou Diarra, Djeneba K. Dabitao, Mamadou Wague, Garan Dabo, Seydou Doumbia, Jane L. Holl, Robert L. Murphy, Souleymane Diallo, Almoustapha I. Maiga, Mamoudou Maiga, Sounkalo Dao

**Affiliations:** 1 University Clinical Research Center (UCRC) Laboratory, University of Sciences, Techniques and Technologies of Bamako (USTTB), Bamako PB 1805, Mali; ycissoko@hotmail.com (Y.C.); issiaka8220@gmail.com (I.C.); okodio@icermali.org (O.K.); mtolofoudie@icermali.org (M.T.); togoacg@icermali.org (A.C.G.T.); sadio@icermali.org (Y.d.S.S.); bbaya@icermali.org (B.B.); seydousamake@icermali.org (S.S.); dialloboubacarib@gmail.com (I.B.D.); bkone@icermali.org (B.K.); amadoukone@icermali.org (A.K.); bdiarra@icermali.org (B.D.); ddabitao@icermali.org (D.K.D.); mwague@icermali.org (M.W.); garandabo@yahoo.fr (G.D.); sdoumbi@icermali.org (S.D.); solo@icermali.org (S.D.); amaiga@icermali.org (A.I.M.); Sounkalod@icermali.org (S.D.); 2 School of Laboratory Medicine and Medical Sciences, University of KwaZulu-Natal, Private Bag X5, Durban 4001, South Africa; 3 Department of Infectious Diseases and Tropical Medicine, Point “G” University Teaching Hospital, Bamako PB 1805, Mali; 4 Institute for Global Health, Northwestern University, Chicago, IL 60611, USA; etienne.dembele@northwestern.edu (E.D.); alishakumar099@gmail.com (A.K.); mohamed.traor12@gmail.com (M.T.); r-murphy@northwestern.edu (R.L.M.); 5 Department of Public Health Sciences, Penn State College of Medicine, Hershey, PA 17033, USA; djibrilba@phs.psu.edu; 6 Department of Neurology and Center for Healthcare Delivery Science and Innovation, University of Chicago, Chicago, IL 60611, USA; jholl@neurology.bsd.uchicago.edu

**Keywords:** SARS-CoV-2, COVID-19, seroprevalence, healthcare workers, Bamako, Mali, West Africa

## Abstract

In Mali, a country in West Africa, cumulative confirmed COVID-19 cases and deaths among healthcare workers (HCWs) remain enigmatically low, despite a series of waves, circulation of SARS-CoV-2 variants, the country’s weak healthcare system, and a general lack of adherence to public health mitigation measures. The goal of the study was to determine whether exposure is important by assessing the seroprevalence of anti-SARS-CoV-2 IgG antibodies in HCWs. The study was conducted between November 2020 and June 2021. HCWs in the major hospitals where COVID-19 cases were being cared for in the capital city, Bamako, Mali, were recruited. During the study period, vaccinations were not yet available. The ELISA of the IgG against the spike protein was optimized and quantitatively measured. A total of 240 HCWs were enrolled in the study, of which seropositivity was observed in 147 cases (61.8%). A continuous increase in the seropositivity was observed, over time, during the study period, from 50% at the beginning to 70% at the end of the study. HCWs who provided direct care to COVID-19 patients and were potentially highly exposed did not have the highest seropositivity rate. Vulnerable HCWs with comorbidities such as obesity, diabetes, and asthma had even higher seropositivity rates at 77.8%, 75.0%, and 66.7%, respectively. Overall, HCWs had high SARS-CoV-2 seroprevalence, likely reflecting a “herd” immunity level, which could be protective at some degrees. These data suggest that the low number of cases and deaths among HCWs in Mali is not due to a lack of occupational exposure to the virus but rather related to other factors that need to be investigated.

## 1. Introduction

The coronavirus disease of 2019 (COVID-19), due to the severe acute respiratory syndrome coronavirus-2 (SARS-CoV-2), is believed to have emerged in Wuhan, Hubei Province in China at the end of December 2019 [1]. The disease spread rapidly all over the world, with millions of deaths reported [2]. The World Health Organization (WHO), on 30 January 2020, declared the outbreak a “public health emergency of international concern” and then, on 11 March 2020, defined it as a pandemic disease. As of 24 December 2021, WHO reported nearly 277 million confirmed cases and more than 5.3 million deaths worldwide from COVID-19 [2]. Although the epicenter of the disease outbreak was first in Asia, then in Europe, and later in the United States of America and South America, the WHO predicted and warned that the African continent could become the next epicenter, with dire consequences due to the weakness of the healthcare systems and limited resources and organization.

Mali, located in West Africa with an estimated population of 21 million people, recorded its first case of COVID-19 on 25 March 2020, and, as of 24 December 2021, less than 20 thousand confirmed cases and 648 deaths related to COVID-19 had been reported [2]. The incidence of cases and deaths is similarly low in the general population of Mali and among healthcare workers (HCWs), many of whom were providing direct care to COVID-19 patients. The mortality rate from COVID-19 in the general Malian population, as of 24 December 2021, was 3.3% [2]; however, there is no specific data available regarding HCWs’ mortality from COVID-19 in Mali. Similar data from the general population have been reported for most countries in sub-Saharan Africa [3]. Yet, HCWs were a high-risk group for SARS-CoV-2 infection, especially in these low-resource countries where personal protective equipment (PPE) was non-existent and stringent infection prevention and control measures were lacking or not adhered to. A recent meta-analysis found that the proportion of SARS-CoV-2-positive HCWs among all positive cases was estimated to be 10% worldwide, with severe cases and deaths being lower [4].

Although many SARS-CoV-2 serological surveys have been performed about HCWs in industrialized countries [4,5,6,7,8,9,10,11], few have reported about HCWs in sub-Saharan countries [12,13,14,15,16,17]. HCWs are supposedly at higher occupational exposure risk and, therefore, need additional layers of protection. A study in the United States focused on HCWs with different levels of exposure to COVID-19 showed that those with more contact with cases had similar seroprevalence rates to those with limited or no occupational exposure [18], possibly suggesting that adherence to PPE use was effective in preventing transmission to HCWs.

We found in this study that the seroprevalence of SARS-CoV-2 is very high in HCWs in Mali. Another study before the COVID-19 vaccine from Mali in the general population, including rural and urban areas, and using a different serology assay found similar rates that we found in HCWs, around 70% of seropositivity. It is likely that the population met the “herd immunity” level, which may explain at least in part the limited numbers of severe cases, although the delta variant is confirmed to be circulating in Mali [19].

## 2. Materials and Methods

The research protocol was approved on 9 September 2020 by the COVID-19 Scientific Committee of Mali (No_06_P/CoS) and the Ethics Committee of the Faculty of Medicine, Pharmacy and Odonto-Stomatology of Bamako of the University of Sciences, Techniques and Technologies of Bamako (USTTB) in Mali (No_2020/194/CE/FMOS/FAPH). All participants signed the approved consent form.

### 2.1. Study Design and Population

This cross-sectional study was conducted between November 2020 and June 2021 at three hospitals in Bamako, the capital city of Mali, that has special COVID-19 treatment units.

HCWs, including physicians, nurses, respiratory therapists, pharmacists, housekeeping, and administrative personnel, were recruited by our clinical research team. The hospital directors were informed of the study through an official letter to inform the HCWs of opened enrollments. HCWs were eligible to take part in the study if they had no symptoms suggestive of COVID-19 on the day of enrollment and had no positive PCR test for COVID-19 in the last three days before study enrollment. However, participants were asked to report all symptoms experienced and PCR results in the past 3 months of study enrollment.

Sociodemographic and clinical data were collected using a brief survey administered to each participant before obtaining a blood sample. A research assistant then entered the survey data into a REDCap database.

A 5 mL venous blood sample was obtained from each participant using a BD Vacutainer™ Venous Blood Collection Tubes, SST™ Serum Separation Tubes (Becton Dickinson, Franklin Lakes, NJ, USA) by a trained phlebotomist. Blood samples were transported to the University Clinical Research Center (UCRC) laboratory, where the serum was extracted, aliquoted, and stored at −80 °C freezer until SARS-CoV-2 IgG level determination.

### 2.2. SARS-CoV-2 IgG ELISA

The SARS-CoV-2 IgG ELISA kit was used (Enzo Life Sciences, Farmingdale, New York, NY, USA), and the manufacturer’s instructions were followed. The SARS-CoV-2 IgG enzyme-linked immunosorbent assay is designed for the qualitative detection of IgG antibodies to SARS-CoV-2 in human serum with a 96-well microplate coated with the SARS-CoV-2 S1 antigen RBD protein. A complex is formed between the SARS-CoV-2 S1 antigen RBD proteins when there is a presence of SARS-CoV-2 IgG in the serum sample. A colorimetric assay is then performed with the intensity of coloration proportional to the amount of anti-SARS-CoV-2 IgG antibodies in the serum of participants.

The SARS-CoV-2 IgG ELISA Kit (ENZ-KIT170-0001) and sera were brought to room temperature. The sera were then heat inactivated at 60 °C for 30 min. Serum samples were diluted with the sample diluent reagent in a 1:10 ratio. In a 96-well microplate, duplicated blank wells were left empty, and 100 µL of high positive control, 100 µL of low positive control, and 100 µL of negative control were loaded in duplicate to the appropriate wells. Then, 100 µL of each diluted sample was loaded into the remaining wells, except the blank wells. Plates were sealed and incubated at 37 °C for 30 min. Contents of wells were aspirated and immediately washed with 350 µL of the wash buffer using a plate washing machine Biotek EL 406 (Biotek, Santa Clara, CA, USA). Thereafter, 100 µL of the HRP conjugate was added to each well except the blank wells and incubated at 37 °C for 15 min. After incubation, the contents of the plate were aspirated and washed with 350 µL of the wash buffer. After the second step, 100 µL of the TMB substrate was added to each well, including the blank wells, and incubated at 37 °C for 15 min in the dark. This reaction was stopped by adding 50 µL of the stop solution. The plate was then read within 15 min after adding the stop solution using a microplate reader, VersaMax^TM^ ELISA Microplate Reader (VersaMax^TM^, Corston, UK) with an optical density of 450 nm.

### 2.3. Validation of the Assay and Determination of Index Values

The ELISA assay was considered valid according to the manufacturer when the mean absorbance (mean optical density units) of the high positive control from the test kit was ≥0.50, the low positive control was ≥0.10, and the negative control was ≤0.08. To calculate the index values, the net OD (optic density) values were first determined as the average OD of the two blank wells subtracted from all OD values, including the negative control, low positive control, high positive control, and samples.

The following formula was used:

(1) Net Sample OD= Sample OD−Average Blank OD

The data comparison between different assay runs was facilitated using an index value whereby sample absorbance was expressed relative to the assay cut-off value (COV). A sample’s index value was calculated using the following formula:

(2) $$\mathrm{Index}\;\mathrm{Value}\;=\frac{\mathrm{Net}\;\mathrm{Sample}\;\mathrm{OD}}{\mathrm{Cut}\_\mathrm{off}\;\mathrm{Value}\;\left(\mathrm{COV}\right)}$$

The manufacturer’s COV was experimentally determined to be 0.08 in the U.S. population, and a sample is considered to be positive if it has an index value greater than one, based on the U.S. population COV. However, this was not applicable to our study population with high background signals [20,21]. Adjustments are generally needed for serology tests in populations with high antibodies/protein background signals where infections are frequent, such as in Mali [20,21]. This corrective strategy is well known and has been used for many serology kits, including for COVID-19 [16]. We, therefore, determined the COV of the Malian population for this kit using serum samples from 2016, well before COVID-19. The few outliers detected in the pre-COVID-19 samples were likely caused by cross-reactivity with coronaviruses (~7% in the Malian population) other than SARS-CoV-2 and are considered to be false positives [21]. Given that the presence or absence of anti-SARS-CoV-2 virus IgG is determined in relation to the calculated COV, a specimen was considered negative when the index value was <3.0, meaning no detection of the SARS-CoV-2 spike protein IgG and implying no prior exposure found and a specimen was considered positive when the index value was ≥3.0, indicating detection of the SARS-CoV-2 spike protein IgG, thus, implying prior exposure to SARS-CoV-2 found.

We considered specimens with an index value <3 as seronegative, specimens with index values ≥3 but <5 as seropositive with low reactivity, and specimens with index value >5 as seropositive with strong reactivity.

### 2.4. Statistical Analysis

Descriptive statistics, including frequencies and percentages, were reported for categorical variables and means for continuous variables. Univariable logistic regression was used to estimate the odds ratio (OR) and 95% confidence intervals (95% CI) for the seropositivity of SARS-CoV-2 with no mask-wearing most of the time being the reference group. Thus, a high risk of SARS-CoV-2 exposure was defined as an HCW who provided direct care to hospitalized COVID-19 patients who had positive SARS-CoV-2 RT-PCR tests. Low risk of SARS-CoV-2 exposure was defined as an HCW who did not provide direct care to hospitalized COVID-19 patients with positive SARS-CoV-2 RT-PCR tests. The trend analysis was conducted using chi-square statistics. For all statistical analyses, significance levels were 2-sided with a *p*-value less than 0.05. Main analyses were conducted using SAS 9.4 (SAS Institute Inc., Cary, NC, USA). The trend analysis was performed with GraphPad Prism (San Diego, CA, USA). Further analyses were performed using SPSS 20.0 (SPSS, Inc., Chicago, IL, USA) to perform Mann–Whitney U test to compare means of delay between SARS-CoV-2 RT-PCR and serology in participants with positive vs. negative PCR within seropositive cases and to perform the Fisher exact test in assessing the association between symptoms in the three months prior to study enrollment, and the serology result of the anti-SARS-CoV-2 IgG.

## 3. Results

### 3.1. Study Population

A total of 240 HCW participants were enrolled in the study, but data for 238 participants were included in the final analysis, as 2 participants had missing information.

### 3.2. Participant Sociodemographic and Clinical Characteristics

**Figure 1:**
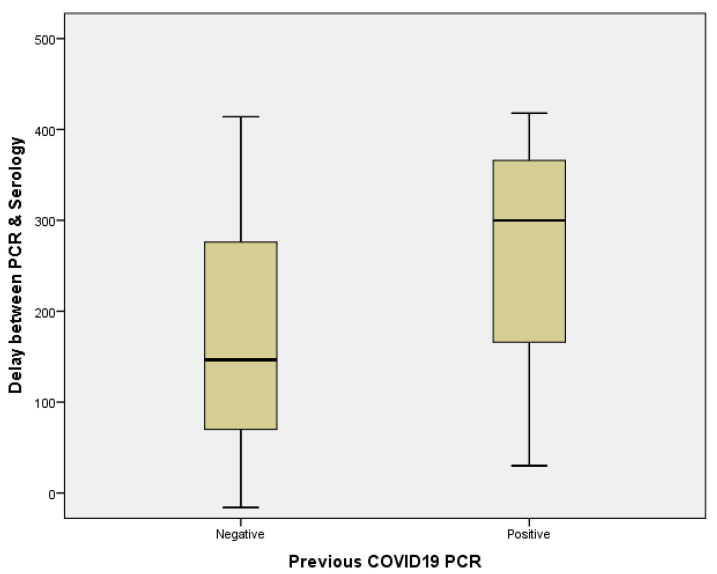
Seroprevalence and clinical characteristics.

Most of the study participants worked in the inpatient hospital setting. Nurses and physicians were the most represented, 22.7% and 18.9%, respectively. Most study participants were males, and the mean age (SD) was 33(9) years. Seropositive participants, despite not having reported any COVID-19 symptoms at enrollment, reported more general symptoms, including sore throat (71.4% vs. 28.6%), fatigue (66.7% vs. 33.3%), headache (69.6% vs. 30.4%), congestion (69.1% vs. 30.9%), and fever (66.7% vs. 33.3%) in the past three months. Among the HCWs, there were those with comorbidities, and the seropositive participants accounted for more comorbidities than seronegative participants, including obesity (87.5% vs. 22.2%), asthma (75.0% vs. 25.0%), and diabetes (66.7% vs. 33.3%) (Table 1). Self-reported information on the PCR test, 35 participants reported that they had been tested for COVID-19 three months prior to study enrollment, and among those with test results, 6 participants tested positive for COVID-19 and 29 tested negative. Statistically significant differences in the time of the result of PCR and study enrollment between these two groups was 297.0 ± 133.7 days for previously tested positive vs. 169.4 128.9 days for previously tested negative (*p* = 0.031, Mann–Whitney U test) (Figure 1).

Among all symptoms self-reported in the three months prior to enrollment, loss of smell (85.7% of loss of smell have positive IgG) and loss of taste (100% of loss of taste have positive IgG) were significantly associated with positive serology for anti-SRAS-CoV2 IgG. Fisher exact test unilateral p respectively equal 0.05 and 0.005.

### 3.3. Impact of Occupational Exposure of SARS-CoV-2, Trends among Healthcare Workers in Bamako, Mali

#### 3.3.1. Self-Reported Occupational Exposure to SARS-CoV-2

Overall, 99.2% of study participants were considered to be at high exposure risk, as they provided direct care to COVID-19 patients and most (83.6%) reported using PPE as recommended. Among those at high exposure risk, 62.8% were found to be seropositive. However, among those classified as low exposure risk, none were seropositive (Table 2).

**Figure 2:**
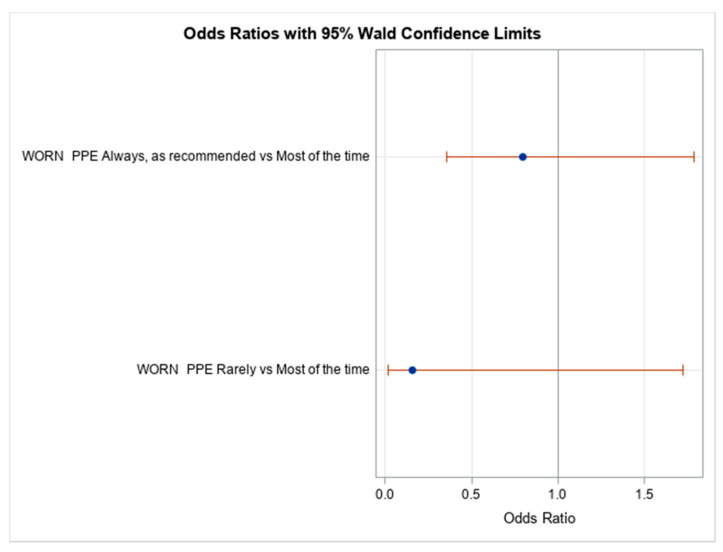
Association between occupational exposure and seroprevalence. Odds ratios of seropositivity in participants with PPE use as recommended and PPE use rarely were compared to those with PPE use most of the time. Odd ratios estimates were calculated using logistic regression.

No statistically significant difference was found between seroprevalence and PPE use as recommended (OR 0.80, 95% CI 0.36–1.79) or rarely use of PPE (OR 0.16, 95% CI 0.02–1.72) (Figure 2).

#### 3.3.2. Seroprevalence Trends Overtime

**Figure 3:**
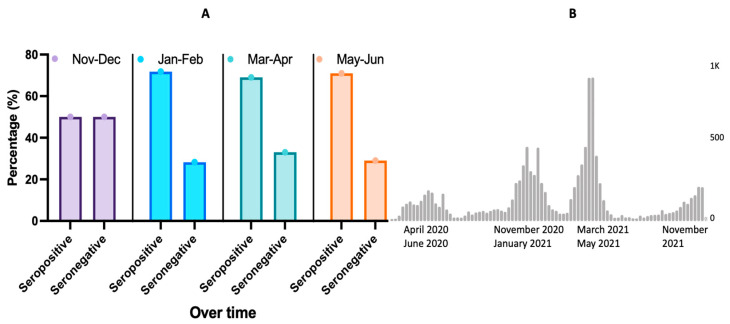
(**A**) Seroprevalence bimonthly trend from November 2020 to June 2021 and (**B**) waves of SARS-CoV-2 cases in Mali.

From November 2020 to December 2020, about 50% of HCW participants were seropositive. However, starting from January 2021, approximatively 70% of HCW participants were seropositive, and this high trend remained stationary from March to June 2021 (Figure 3A).

**Figure 4:**
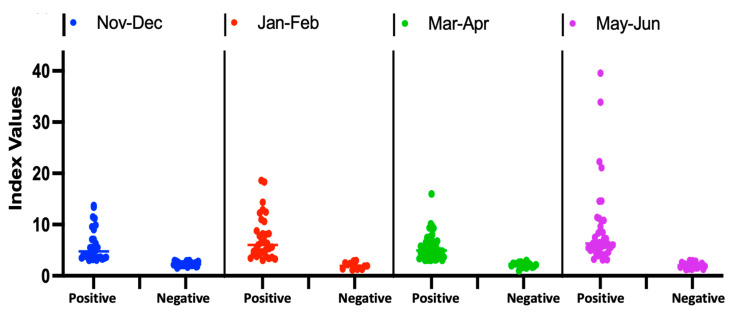
Level of SARS-CoV-2 spike protein IgG in Mali over time.

Of note, the two biggest waves occurred during the study period, with the first big wave from November 2020 to January 2021 and the second biggest wave from March 2021 to May 2021 (Figure 3B) [2]. The level of SARS-CoV-2 spike protein IgG (based on index values) increased during the course of the COVID-19 pandemic, with the highest index value (39) reported toward the end of our study (May–June) (Figure 4).

## 4. Discussion

We found a high seroprevalence of SARS-CoV-2 IgG among HCWs at three hospitals providing care to COVID-19 patients in Mali, despite low numbers of reported COVID-19 cases and deaths in the general population. Seroprevalence increased over time from 50% in October 2020 to 70% in June 2021, following two waves of the pandemic in Mali (Figure 3). In another seroprevalence study before the COVID-19 vaccine from Mali in the general population, seropositivity of SARS-CoV-2 IgG was also high at 58.5% in rural areas and 73.4% in urban areas [16]. On the one hand, it is likely that the population met the “herd” immunity level, which may explain at least in part the limited numbers of severe cases, although the delta variant is confirmed to be circulating in Mali [19]. Whether or not natural immunity is sufficient to protect against further infection and disease still remains to be determined.

This study nevertheless reveals a high and increasing trend of seroprevalence over time, highlighting perhaps the ineffectiveness or lack of adherence to mitigation measures. The predictions by WHO and other experts at the beginning of the pandemic that exposure to the SARS-CoV-2 will be important is proven to be true, although fortunately, the number of disease cases and deaths did not follow the trend seen in other settings. It is reasonable to assume that the low number of confirmed cases and related deaths may not reflect the reality of the pandemic in Mali, given the limited resources and poor healthcare infrastructure. Of note, thus far, only 27% of HCWs in Africa received full doses of COVID-19 vaccination [22]. This low rate of vaccination further exposes these workers to a higher risk. However, deaths, even if under-reported, are still not of the same magnitude as in industrialized countries. If 3% of COVID-19 cases had died in Mali, we should have seen many more cases of unexplained deaths, whether confirmed or assumed not to be linked to COVID-19.

Many factors may explain why symptomatic COVID-19 and related deaths are relatively low in Mali, despite high seroprevalence among HCWs and the general population. The lower seed rate of SARS-CoV-2 in rural areas where approximately 57% of the population lives [23] and the young mean age of the population with 40% estimated to be less than 14 years old are potentially key factors. Even the HCWs study population, which only included individuals >18 years of age, had a mean age of only 33 years (this is representative of the HCWs’ population in Mali). Lifestyle may also contribute, given the sub-Saharan climate in Mali with many activities of daily living (e.g., cooking, eating, sleeping) being conducted outdoors. Other potential factors, such as the virulence level of circulating variants, frequent exposure to other pathogens, particularly other coronaviruses, and the resulting maturity of the immune system, and the role of the microbiome and hygiene hypothesis [24], need to be carefully examined.

Interestingly, there was no association between level of occupational exposure and seroprevalence (Figure 2), implying that working in a hospital environment, whether at high or low occupational exposure risk, did not seem to affect seroprevalence. HCWs belong to the same community as the rest of the general population and seem to have the same level of exposure as everyone else in the community. They are more conscious of the risks associated and, thus, take more protective measures in their work setting. Our study differs from other studies that found that seroprevalence was higher among HCWs, but is consistent with studies that found higher seroprevalence in urban areas that have greater population density [11,13,16]. This study is also consistent with a recent SARS-CoV-2 seroprevalence study conducted in Cameroon [17], which reported that at least one COVID-19 related symptom was seen among the seropositive individuals. HCWs are more likely to report they were wearing PPE as recommended. The self-report of both anosmia (loss of smell) and ageusia (loss of taste) among seropositive HCWs, although not recognized as symptoms of COVID-19 at the beginning of the pandemic, further confirms their significance as predictive symptoms for COVID-19. A multicentric study in Europe found 85.6% and 88.0% loss of smell and loss of taste, respectively, among COVID-19 patients [25]. Moreover, a recent study found SARS-CoV-2 seroprevalence to be positively associated only with persistent anosmia (odds ratio, 2.72; 95% CI, 1.66–4.46) [26]. In our study, these two symptoms were significantly associated with the seropositivity of HCWs in Mali.

The majority of the HCWs were exposed to SARS-CoV-2, among whom there were participants with comorbidities, which could increase the risk of severe forms in the case of COVID-19. This group of HCWs with comorbidities should perhaps be exempted from providing direct care to COVID-19 patients; otherwise, the protection measures should be enhanced.

This study has some limitations. The same study participants were not followed over time, and therefore, we cannot comment on subsequent symptoms, COVID-19 disease cases, or related deaths. Information about prior exposures, including but not limited to symptoms and use of PPE, was self-reported, which is prone to bias. Nevertheless, the study revealed several important findings that help to understand the trends of the pandemic in this region, where reported cases and deaths are relatively low.

## 5. Conclusions

This study about seroprevalence in HCWs in a West African county revealed important and unexpected high seropositivity to SARS-CoV-2 among frontline HCWs, which may help to understand the surprisingly low number of COVID-19 cases and related deaths in Mali and in other similar settings in Africa. This study shows that prior exposures to SARS-CoV-2 were high among HCWs and increased during the two most important waves in the country. The high seroprevalence rate, however, was not proportionate to rates of COVID-19 confirmed cases in Mali. Clearly, further investigation of the many hypothesized factors contributing to high seroprevalence, but lower rates of disease is needed. Studies to determine if the degree to “herd” immunity in the Malian population is protective against emerging coronavirus variants are urgently required, particularly given the highly limited vaccine availability and operational and logistical difficulties to vaccinate the population, even when available.

## Figures and Tables

**Table 1:** Demographics and symptomatology of seropositive and seronegative participants.

Characteristics	All	Seronegative	Seropositive
	*n* = 238	*n* = 91	*n* = 147
Age (years), mean (+/−SD)	33 (9)	34 (9)	33 (9)
Female, *n* (%)	98 (41.2)	35 (35.7)	63 (64.3)
Male, *n* (%)	140 (58.8)	56 (40.0)	84 (60.0)
Profession *n* (%)			
Physicians	45 (18.9)	19 (42.2)	26 (57.8)
Physician assistant	4 (1.7)	2 (50.0)	2 (50.0)
Nurse	54 (22.7)	17 (31.5)	37 (68.5)
Environmental service	15 (6.3)	7 (46.7)	8 (53.3)
Transportation technician	18 (7.1)	9 (50.0)	9 (50.0)
Other	94 (39.1)	31 (33.3)	63 (67.0)
Practice Setting *n* (%)			
Hospital setting	229 (96.2)	91 (39.7)	138 (60.3)
Outpatient setting	12 (5.0)	2 (16.7)	10 (83.3)
Community setting	5 (2.1)	0 (0.0)	5 (100.0)
Symptoms past 3 months *n* (%)			
Fever	66 (28.2)	22 (33.3)	44 (66.7)
Congestion	55 (8.4)	17 (30.9)	38 (69.1)
Sore throat	21 (8.8)	6 (28.6)	15 (71.4)
Dry cough	29 (12.2)	12 (41.4)	17 (58.6)
Headache	79 (33.2)	24 (30.4)	55 (69.6)
Tiredness	72 (30.3)	24 (33.3)	48 (66.7)
Pain	67 (28.2)	25 (37.3)	42 (62.7)
Comorbidities *n* (%)			
Obesity	18 (7.6)	4 (22.2)	14 (77.8)
Diabetes	3 (1.3)	1 (33.3)	2 (66.7)
High blood pressure	4 (1.7)	3 (75.0)	1 (25.0)
Asthma	4 (1.7)	1 (25.0)	3 (75.0)
Smoking	17 (7.1)	8 (47.1)	9 (52.9)

**Table 2:** Occupational exposure to SARS-CoV-2.

Exposure and PPE Use *n* (%)	All	Seronegative	Seropositive
	*n* = 238	*n* = 91	*n* = 147
Higher exposure risk	234 (99.2)	87 (37.2)	147 (62.8)
Lower exposure risk	4 (1.7)	4 (100.0)	0 (0.0)
PPE use			
As recommended	199 (83.6)	74 (37.2)	125 (62.8)
Most of the time	31 (13.0)	10 (32.3)	21 (67.7)
Rarely	4 (1.7)	3 (75.0)	1 (25.0)
